# Development of a novel assay for antigen presentation measurement

**DOI:** 10.1038/s41598-025-13997-y

**Published:** 2025-09-26

**Authors:** Mei Li, Falak Harshit Sharma, Yi-Ling Chen, Marco Esteban Araneda, Amy Hammett, Derick Miller, Lilly Pearce, Kuan-Hui E. Chen

**Affiliations:** 1 Department of Biological Sciences, Texas Tech University, Lubbock, TX 79409 USA; 2 Department of Electronic Engineering, National Kaohsiung University of Science and Technology, Kaohsiung, 80778 Taiwan

**Keywords:** Antigen presentation, MHC I/II, Click chemistry, Phagocytosis, Breast cancer, Biological techniques, Biotechnology

## Abstract

Accurate measurement of antigen presentation is essential for understanding immune responses to infections and tumors. However, current methods are cumbersome, time-consuming, and rely on known peptide sequences and antibodies, leading to unstable antigen presentation, antigen loss during processing and editing, and inconsistent results. We developed a novel, cost-effective method for examining antigen presentation using Click chemistry, which utilizes a bioorthogonal reaction between azides and alkynes/cyclooctenes. Antigens were pre-labeled with azides or alkynes to facilitate their uptake by antigen-presenting cells (APCs). Their presentation was subsequently detected using fluorophore-conjugated dibenzocyclooctyne or azide. The study involved three types of APCs, mouse macrophages (RAW264.7), mouse dendritic cells (DC2.4), and mouse primary bone marrow derived dendritic cells (BMDCs), and three categories of antigens: BSA, bacteria, and tumor antigens. Antigen presentation was measured and validated through multiple analytic techniques, including a fluorescent plate reader, flow cytometry, and ELISA. We showed efficient and stable presentation of antigens on the surface of all RAW264.7, DC2.4 and BMDCs. Antigens labeled using Click chemistry showed enhanced stability within the phagolysosomes of APCs. Notably, antigens labeled throughout the peptide sequence using azidohomoalanine (AHA) exhibited superior presentation on MHC class II compared to antigens labeled only at the N-terminus. Furthermore, this method preserved the natural antigen editing process, enabling the selection of high-affinity antigens for MHC presentation. This novel antigen presentation assay offers key advantages over existing methods, including faster processing, cost-effectiveness, stable antigen presentation, and reliable detection signals. When paired with mass spectrometry, it can identify stably presented tumor peptides, offering potential targets for immunotherapy development.

**Supplementary Information:**

The online version contains supplementary material available at 10.1038/s41598-025-13997-y.

## Introduction

Cancer is a complex and multifactorial disease that arises from a combination of genetic, metabolic, endocrinological, and immunological abnormalities^1,2^. Each tumor cell is unique and evolves independently through the accumulation of mutations across various gene loci, a phenomenon referred to as tumor heterogeneity^3^. This inherent diversity among tumor cells poses significant challenges for effective cancer treatment. Recent advancements in cancer therapy have focused on targeting specific tumor-associated antigens, enabling more precise and personalized approaches to treatment^4–6^. However, tumor cells exhibit a remarkable ability to adapt and develop resistance to these therapies. One common mechanism of resistance involves the reduction or loss of the targeted tumor antigens^7^, which allows the cancer cells to evade therapeutic interventions and continue to proliferate. Additionally, reduced MHC expression by cancer cells has also been reported^8,9^. This adaptive resistance highlights the need for the continued development of innovative strategies to overcome the dynamic and resilient nature of cancer.

T cells represent the primary immune cell population responsible for targeting and eliminating tumor cells. However, priming effector T cells to effectively attack tumors is a complex and tightly regulated process. Naïve T cells must first undergo activation, a process initiated by antigen-presenting cells (APCs). These APCs are critical intermediaries that “educate” naïve T cells by presenting tumor-derived antigens via major histocompatibility complex (MHC) molecules^10^. This interaction is essential for the differentiation of naïve T cells into fully functional effector T cells capable of recognizing and destroying tumor cells. Consequently, the efficiency and fidelity of antigen presentation directly influence the strength and specificity of the immune response against cancer, highlighting its significance in developing therapeutic strategies.

Antigen presentation is essential for activating adaptive immunity and occurs via MHC class I and II pathways. MHC class I primarily presents endogenous antigens, such as viral or cytosolic proteins^11^. In APCs, these proteins are degraded by the ubiquitin–proteasome system (UPS) into peptides, which are then transported into the endoplasmic reticulum (ER) by TAP^11^. In the ER, peptides bind to newly synthesized MHC I molecules, a process regulated by ERp57, which removes low-affinity peptides through antigen editing^12^. The resulting peptide–MHC I complexes are transported to the cell surface for recognition by CD8⁺ T cells. The MHC class II pathway primarily presents extracellular antigens^13^. After uptake by APCs via endocytosis or phagocytosis, these antigens are degraded into peptides within endosomes. MHC class II molecules, synthesized in the ER, are transported to the endosomal compartments to load the peptides^13^. The resulting peptide–MHC II complexes are then displayed on the cell surface for recognition by CD4⁺ T cells. Additionally, certain extracellular antigens within endosomes can be presented via MHC class I through a process known as cross-presentation^14^. This can occur either by exporting the antigens from endosomes into the cytosol^15^, where they enter the conventional MHC I pathway, or through the reported import of MHC I molecules into endosomes^16^. Moreover, recent studies highlight the role of autophagy in antigen presentation by delivering cytosolic antigens to lysosomes for MHC class II loading^17^ or enhancing cross-presentation via the MHC class I pathway^18^.

However, measuring antigen presentation currently remains a significant challenge due to its labor-intensive and time-consuming nature. A typical approach requires the pre-definition of an antigen peptide sequence, followed by the use of highly specific antibodies to detect the surface presentation of that antigen on MHC molecules with flow cytometry or immunochemistry staining^19–23^. However, if the pre-defined antigen does not meet all these requirements, it may result in weak interactions with MHC molecules. Further complicating this process is the common tumor immune evasion strategy of downregulating MHC expression, which undermines antigen detection. This raises concerns about the reliability of antigen presentation, particularly when the peptide has weak MHC binding and the expression of MHC molecules is low. In such cases, the presentation may become unstable, leading to inconsistent detection and experimental results. Therefore, while such an approach offers a targeted means of detection, it is inherently limited by the dependence on prior knowledge of the peptide repertoire. Alternatively, mass spectrometry has emerged as a powerful tool for analyzing peptide presentation on the cell surface^24^. This technique provides a broader view of the peptides bound to MHC molecules. However, despite its advanced capabilities, mass spectrometry also has limitations, as it requires sophisticated instrumentation and extensive data analysis. Furthermore, it faces challenges in differentiating between peptides that successfully bind to MHC molecules and those that are processed but fail to achieve stable presentation.

**Fig. 1:**
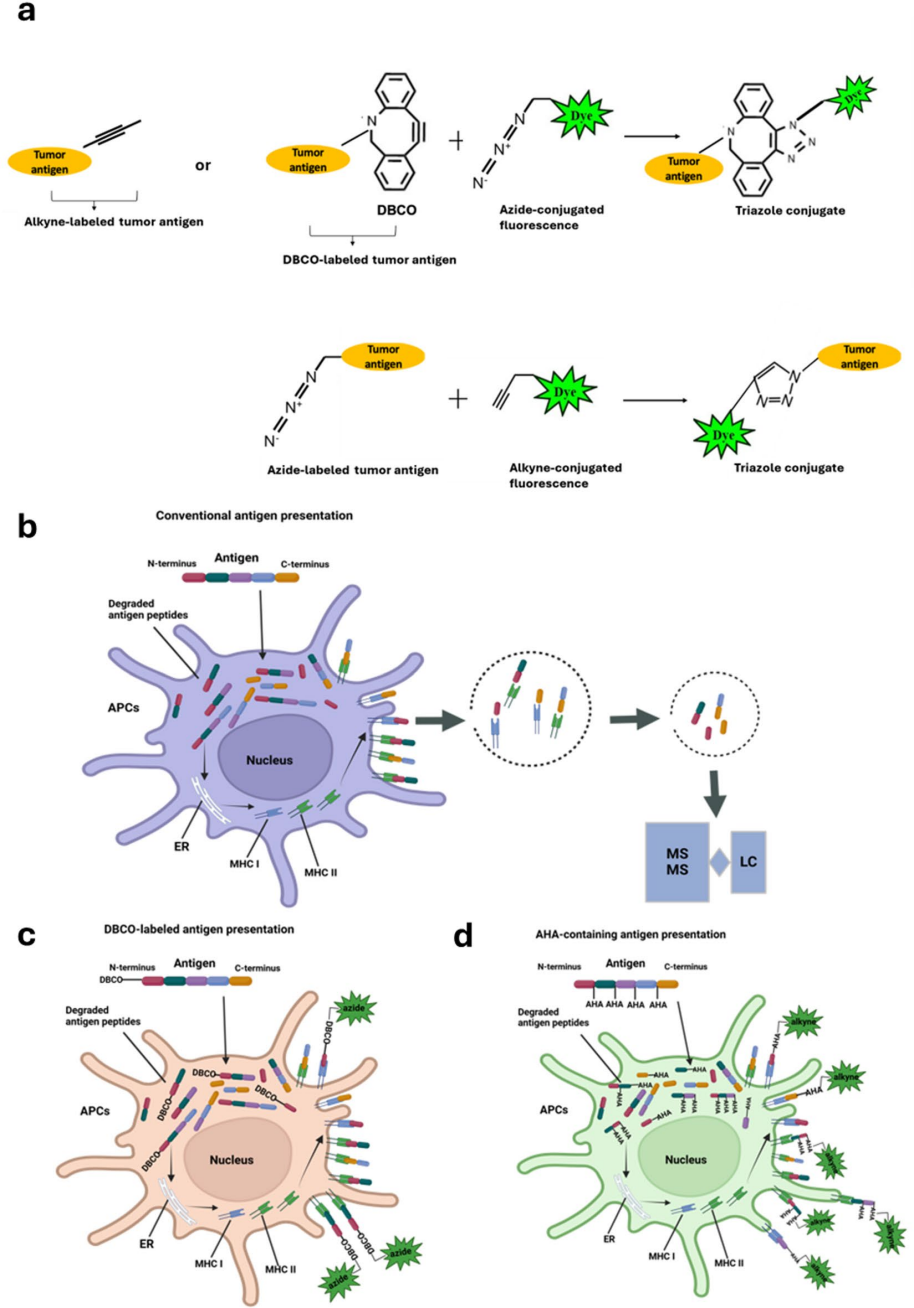
Chemical scheme outlining the click-chemistry use in this study and comparison of conventional and our novel antigen presentation assays. Click-chemistry is a reaction between azides and alkynes/cyclooctenes. Thus, tumor antigens labeled with DBCO or azide can be detected using fluorescent probes conjugated with azide or alkyne, respectively (**a**). The stability and reliability of antigen presentation in conventional assays heavily depend on the sequence of the predefined antigen (**b**). Terminal labeling with DBCO across diverse antigens enables APCs to select the antigen(s) that best fit MHC molecules but introduces the risk of N-terminal truncation during antigen processing (**c**). In contrast, integrating AHA throughout the entire antigen across diverse antigens increases the likelihood that click signals remain detectable in the final presented antigens, regardless of how the antigens are processed and selected (**d**).

Click Chemistry was first introduced by K. Barry Sharpless in 2001^25^. Then this field was significantly advanced by Carolyn Bertozzi^26,27^ and is a reaction between azide and cyclooctyne (or alkyne) components to form a stable 1,2,3-triazole linkage^28^. Because both azide and cyclooctyne groups are not present in any biomolecules, it therefore reduces the non-specific reactions to any cellular components. Thus, the click chemistry is extensively used by biological systems to detect nascent protein synthesis, and it is safe, stable, rapid, and efficient^29^. Taking advantage of this, we therefore develop a new and cost-effective method for analyzing antigen presentation using click chemistry. This is achieved by employing various fluorophore-conjugated azides, alkynes, or cyclooctenes, to interact with alkyne/azide-labeled antigens on the cell surface (Fig. 1a). A comparison of conventional and our novel antigen-presenting assay is shown in Fig. 1. In a conventional antigen presentation assay, a predefined antigen is taken up by antigen-presenting cells (APCs) through phago-/endocytosis. The antigen is then digested and loaded onto MHC II molecules in endosomes, which are transported to the cell surface for presentation. Alternatively, some of the predefined antigen may be transferred to the cytosol, where it undergoes degradation by the ubiquitin-proteasome system (UPS). These degraded fragments can then be transported into the endoplasmic reticulum and loaded onto MHC I molecules for cross-presentation. Because the predefined antigen serves as the sole source of antigens, the interaction strength between the MHC and the antigen is highly dependent on the sequence of the predefined antigen (Fig. 1b).

Using tumor antigens as an example, we propose labeling all tumor peptides with click molecules, either through N-terminally labeled DBCO (Fig. 1c) or by conjugation with azidohomoalanine (AHA) across the entire peptide sequence (Fig. 1d). This approach maximizes antigen diversity, increasing the likelihood of selecting the most effective antigen for presentation during the antigen-editing process. Additionally, AHA conjugation, which labels the entire peptide, mitigates concerns about losing terminal labeling during degradation by the UPS system, resulting in more stable signals. The DBCO-labeled and azido-integrated antigenic fragments on the surface of APCs can be detected using fluorophore-conjugated azide and alkyne, respectively.

In this study, we present a novel methodology for examining antigen presentation. Our approach overcomes the limitations of existing antigen presentation assays and offers greater practicality for analyzing heterogeneous antigens.

## Results

### Antigens modified with DBCO or AHA did not affect APC recognition or subsequent phagocytosis

**Fig.2:**
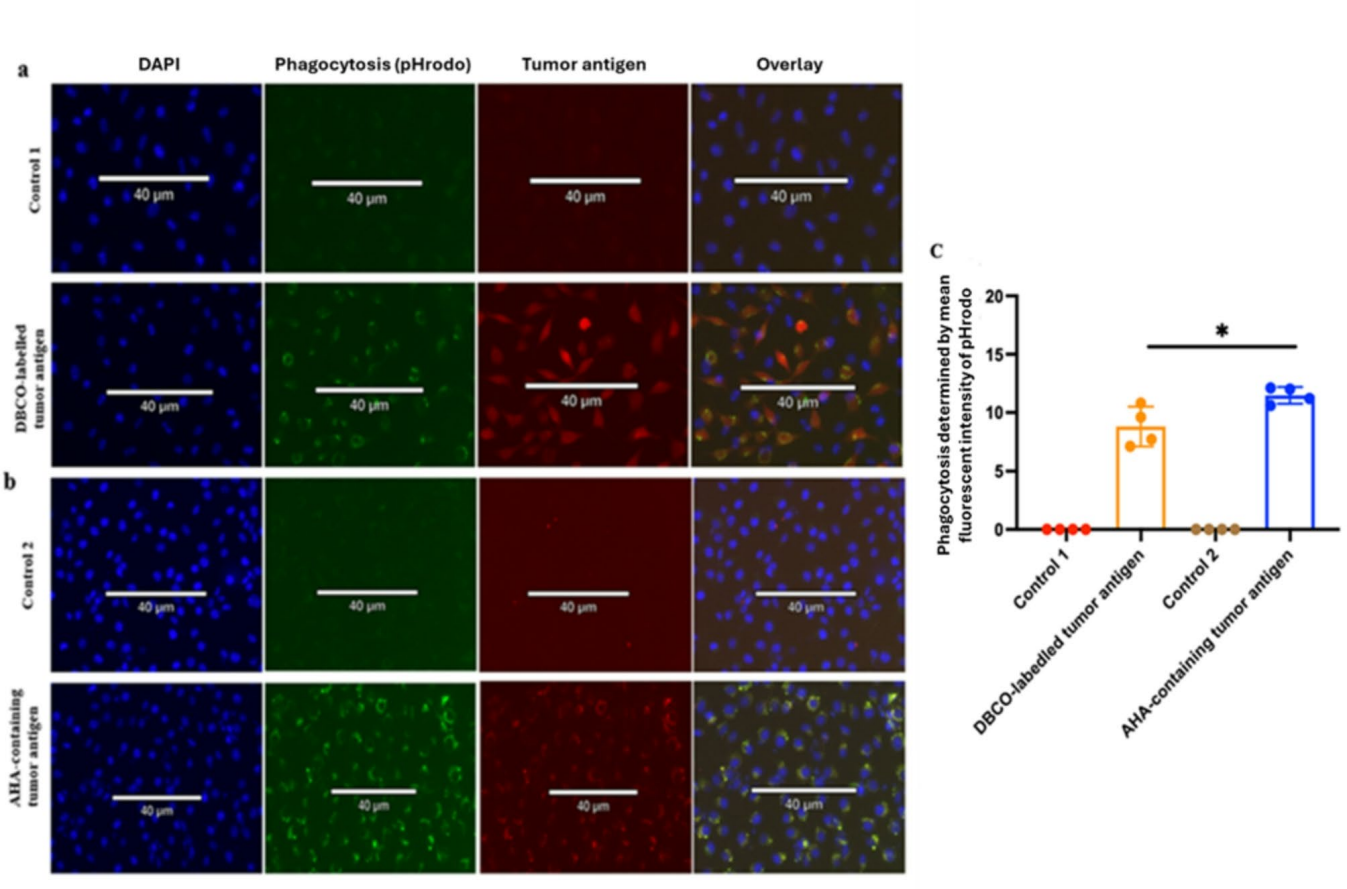
Azido- and alkyne-labeled amino acids demonstrate stability within phagolysosomes. Triple immunofluorescence staining was performed to evaluate the phagocytosis of DBCO-labeled tumor antigens (**a**) and AHA-containing tumor antigens (**b**) by macrophages. Blue fluorescence marks the nucleus, green fluorescence labels the phagolysosome, and red fluorescence identifies the DBCO-labeled or AHA-containing tumor antigens. Macrophages treated without tumor antigens served as controls. The mean fluorescence intensity of the green signal, indicative of phagocytosis, was quantified (**c**). Data are shown as mean ± SD. (*, *p* < 0.05). DBCO, dibenzocyclooctyne; AHA, L-Azidohomoalanine.

APCs primarily rely on pattern recognition receptors (PRRs) which detect conserved molecular structures known as pathogen-associated molecular patterns (PAMPs) and damage-associated molecular patterns (DAMPs), allowing APCs to identify and initiate innate immune responses and activate subsequent adaptive immune responses to invading pathogens or cellular damage. To evaluate whether DBCO- or AHA-modified antigens affect APC recognition, we performed phagocytosis assays using pHrodo dyes and RAW264.7. As seen in Fig. 2a, 0.1 mg of DBCO-conjugated tumor antigens were labeled with pHrodo-green and red fluorescence-conjugated azide that specifically bound to DBCO. At neutral pH, pHrodo Green is non-fluorescent, resulting in the pHrodo Green-DBCO-tumor antigens appearing red due to the reaction of DBCO and red fluorescent-azide. Upon phagocytosis into the acidic environment of the phagolysosome, pHrodo Green became fluorescent, emitting green light. This led to the formation of a stable triazole compound, producing a yellow color. Similarly, AHA-integrated tumor antigens were labeled with pHrodo green and a red fluorescence-conjugated alkyne that specifically reacted with AHA (Fig. 2b). At neutral pH, these antigens appeared red due to the reaction of AHA and red fluorescent-alkyne. However, once in the phagolysosomes, the acidic conditions activated the pHrodo Green fluorescence, resulting in the formation of a yellow triazole signal (Fig. 2b). Our findings demonstrated that N-terminal labeling of antigens with DBCO or internal integration of AHA did not affect antigen phagocytosis by APCs (Fig. 2a and b). Quantitative analysis confirmed efficient internalization of both DBCO- and AHA-modified antigens via phagocytosis (Fig. 2c), with similar findings observed in DC2.4 cells (Supplementary Fig. 1a-c).

### DBCO labeled antigens might be removed during the antigen editing process

**Fig. 3:**
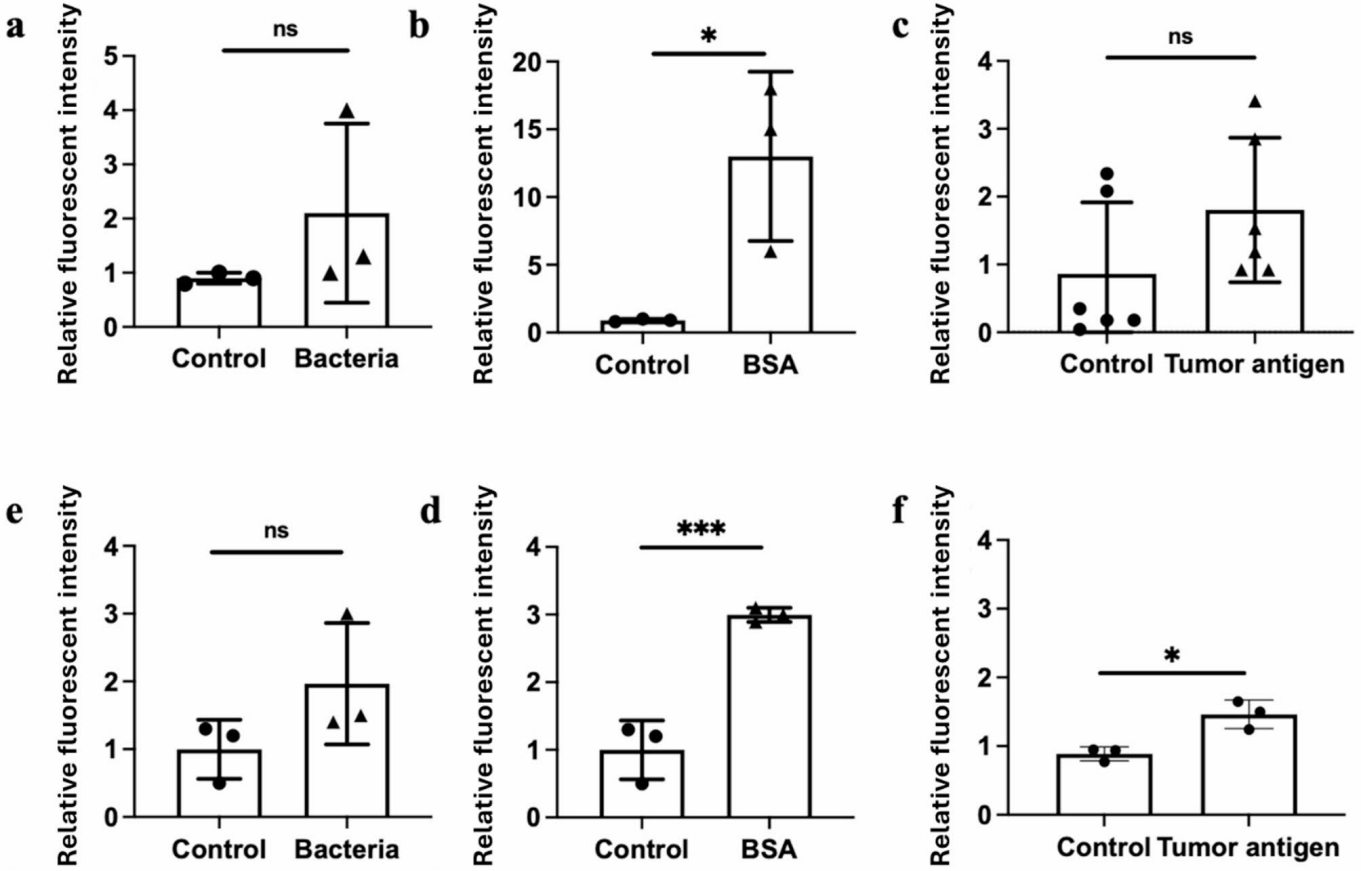
Antigen presentation of DBCO-conjugated antigens by APCs. Mouse macrophages (**a**–**c**) and mouse dendritic cells (**d**–**f**) were cultured with N-terminally DBCO-labeled bacterial antigens (E. *coli*), BSA, and tumor antigens for six days. Surface presentation of DBCO-labeled antigens was detected using the membrane-impermeable azide–Alexa Fluor 488 dye. The resulting fluorescence intensity reflected the surface display of DBCO-labeled E. coli, BSA, and tumor antigens. Untreated cells served as controls. Data are shown as mean ± SD. (*, *p* < 0.05; ***, *p* < 0.001; ns, no statistical difference).

DBCO interacts with amines, leading to its attachment at the N-terminus of peptides. This raised concerns about the antigen presentation of N-terminally labeled peptides to MHC molecules, particularly MHC I, since the Ubiquitin–proteasome system removes terminal amino acids unless they are hydrophobic. To assess whether DBCO-conjugated antigens could lead to stable signals, we treated mouse RAW264.7 and mouse DC2.4 using three DBCO-labelled N-terminus proteins, including *E. coli* protein extracts, BSA, and tumor protein extracts. After incubating for 6 days, impermeable azide-Alexa fluor 488 dye was added to RAW264.7 and DC2.4. The relative fluorescence intensity reflecting surface presentation of DBCO-labeled E. coli, BSA, and tumor antigens was then measured. In RAW264.7, while a trend toward positive signals for antigen presentation via DBCO was observed, it was not statistically significant for either bacterial or tumor antigens (Fig. 3a and c). BSA was the only antigen to show significant results (Fig. 3b), likely due to its simplicity compared to the more significant heterogeneity of bacterial and tumor antigens. A similar pattern was observed in DC2.4, where positive signals for surface DBCO-conjugated antigen presentation were detected. However, significance was not achieved for bacterial antigens (Fig. 3d) and was only marginally met for tumor antigens (Fig. 3f). In contrast, the homogenous DBCO-BSA antigens demonstrated significant results (Fig. 3e).

Collectively, these findings suggest that the new antigen-presenting assay using DBCO conjugation may lack the sensitivity required to reliably detect surface antigen presentation, particularly for more heterogeneous antigens.

### AHA integration provided a more accurate detection of antigen presentation from heterogeneous samples

**Fig. 4:**
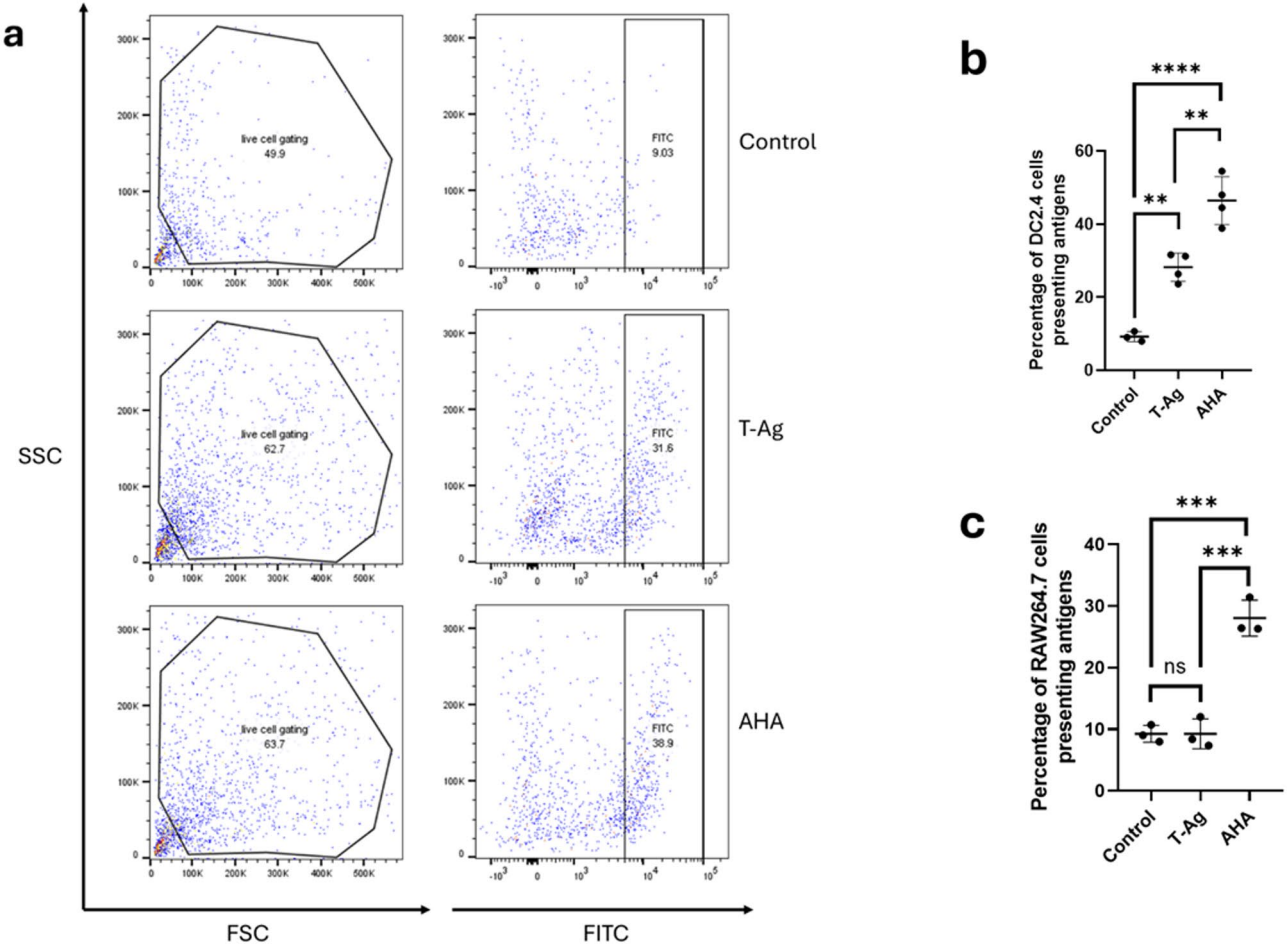
Flow cytometry plots and graphs comparing the surface presentation of fluorescent DBCO- and AHA-labeled tumor antigens by APCs. (**a**) Representative flow cytometry gating strategy showing antigen presentation in dendritic cells untreated (top), treated with DBCO-labeled tumor antigens (T-Ag, middle), and treated with AHA-containing tumor antigens (AHA, bottom). (**b**–**c**) Histograms illustrating the effectiveness of antigen presentation by dendritic cells (**b**) and macrophages (**c**) for DBCO-labeled and AHA-containing tumor antigens. Data are shown as mean ± SD. (**, *p* < 0.01; ***, *p* < 0.001; ****, *p* < 0.0001; ns, no statistical difference).

Since DBCO conjugates exclusively to the N-terminus, which may be removed during antigen editing, we aimed to develop an alternative method that allows the incorporation of click chemistry molecules throughout the entire antigen peptide. AHA, an amino acid analog of methionine, contains a small azido moiety that can be incorporated into proteins during protein synthesis when fed to cultured cells. To evaluate whether AHA integration enhances antigen presentation, we cultured tumor cells in the presence of AHA and collected AHA-integrated tumor peptides for analysis. For comparison, we also included DBCO-conjugated tumor antigens. Theoretically, following the uptake, digestion, and processing of these antigens—within phagolysosomes for MHC II and the ER for MHC I—AHA integration should result in a higher proportion of tumor peptides retaining the azido moiety for click chemistry detection compared to DBCO-labeled tumor peptides. Figure 4A illustrates the flow cytometry gating strategy and provides an example of a comparison of antigen presentation between DBCO-labeled and AHA-labeled tumor antigens. In the representative flow cytometry data, 9.03% of DC2.4 cells in the control group (top), 31.6% in the DBCO-labeled tumor antigen group (middle), and 38.9% in the AHA-containing tumor antigen group (bottom) showed surface fluorescence. Bar graphs summarizing replicate experiments with DC2.4 and RAW264.7 were shown in Fig. 4b and c. The AHA-containing tumor antigen group demonstrated significantly higher surface antigen presentation in DC2.4 (Fig. 4b) and RAW264.7 (Fig. 4c) than the control and DBCO-labeled tumor antigen groups. Notably, only DC2.4 showed significant surface presentation of DBCO-labeled tumor antigens, whereas RAW264.7 did not.

In summary, these results strongly indicate that the AHA incorporation method enhances the efficiency of measuring antigen presentation by APCs.

### AHA-integrated antigens, but not DBCO-conjugated antigens, can be presented on the surface of APCs by both MHC I and MHC II

**Fig. 5:**
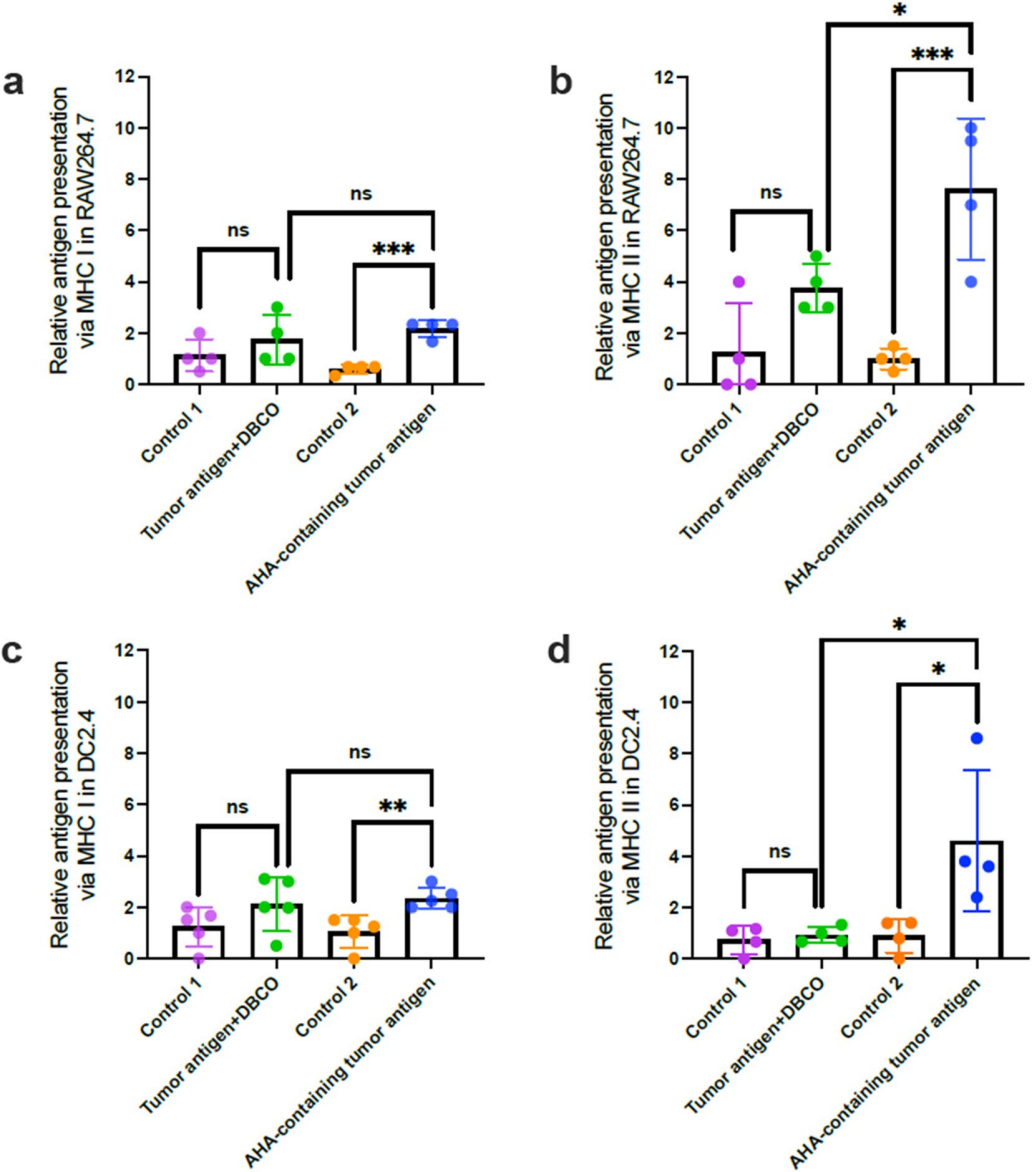
Extracellular DBCO- and AHA-labeled tumor antigens are predominantly presented on MHC class II, with notable cross-presentation on MHC class I. RAW264.7 (**a**–**b**) and DC2.4 (**c**–**d**) cells were cultured with DBCO-labeled or AHA-containing tumor antigens for six days. After fixation and cross-linking, MHC I– or MHC II–presented antigens were captured on plates pre-coated with corresponding antibodies. Antigen presentation was detected via click chemistry using azide- or alkyne-linked HRP, and quantified based on HRP-derived absorbance. Data are presented as mean ± SD. (*, *p* < 0.05; **, *p* < 0.01; ***, *p* < 0.001; ns, no statistical difference).

Given the better antigen-presenting detection in APCs with AHA-containing tumor antigens, as shown in Fig. 4, we hypothesized that AHA-containing tumor antigens can be stably presented on MHC class I and MHC class II. To test this, RAW264.7 and DC2.4 were exposed to tumor antigens labelled with either DBCO or AHA. The cells were then fixed and crosslinked, and protein lysates were analyzed using a sandwich ELISA to assess the presentation of DBCO- or AHA-containing tumor antigens via MHC I and II. The results showed significant antigen presentation of AHA-containing tumor antigens on MHC I (Fig. 5a) and MHC II (Fig. 5b) in RAW264.7. In contrast, DBCO-conjugated tumor antigens showed no significant presentation on either MHC I or II. Similar findings were observed in DC2.4, where AHA-containing tumor antigens were stably crosslinked to MHC I (Fig. 5c) and MHC II (Fig. 5d), whereas DBCO-conjugated tumor antigens did not show stable presentation on either molecule.

In summary, these findings indicate that thoroughly labeled tumor antigens enable consistent and stable measurement of antigen presentation. Moreover, while MHC class II presentation was predominant for these phagocytosed tumor antigens, a significant portion was also cross-presented via MHC class I.

### AHA-incorporated tumor antigens can also be presented by primary mouse bone marrow-derived dendritic cells

**Fig. 6:**
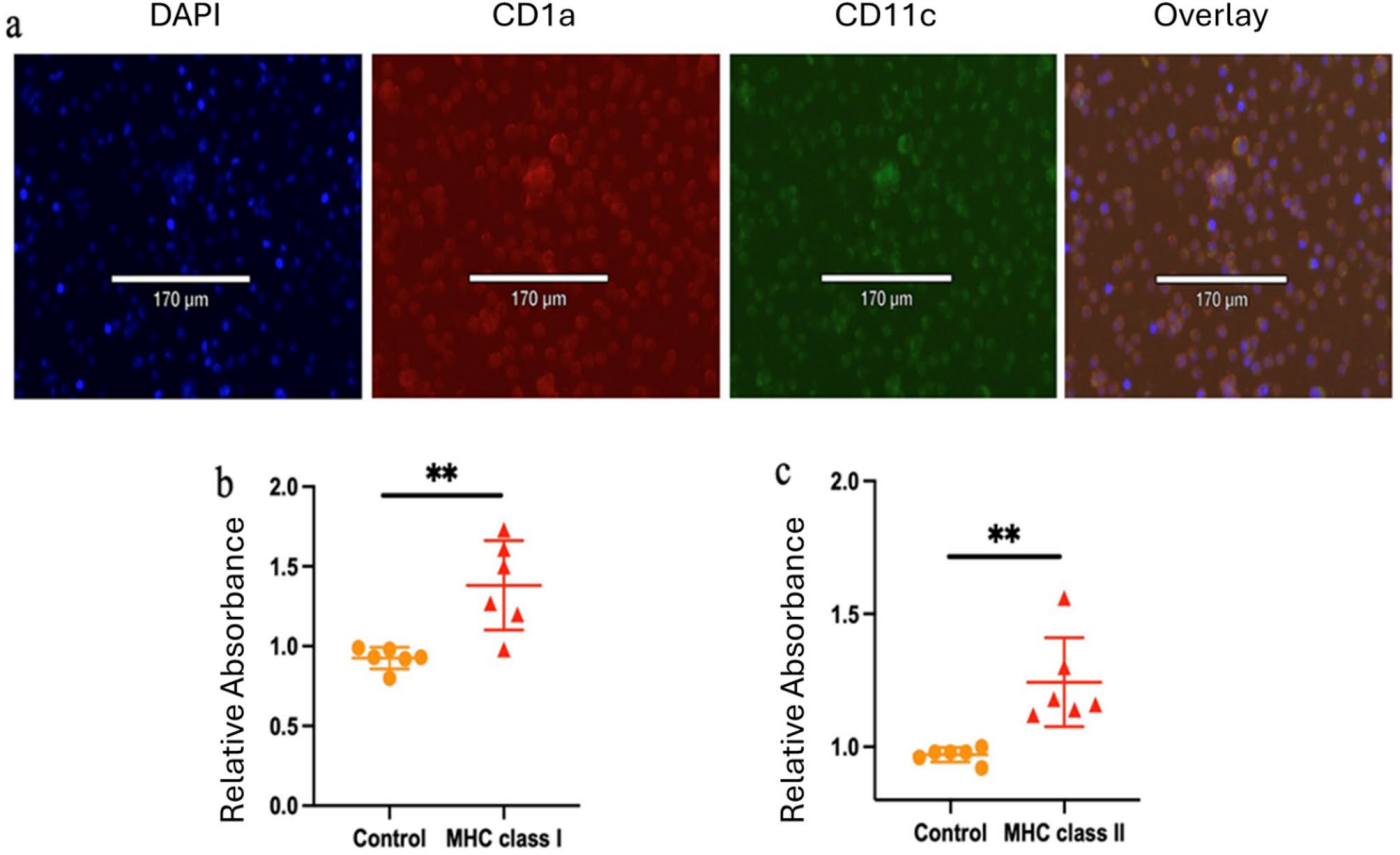
Evaluation of AHA-incorporated tumor antigen presentation via MHC I and MHC II in primary dendritic cells derived from mouse bone marrow. (**a**) Dendritic cell identity was confirmed using CD1a and CD11c (both known as dendritic cell markers). Antigen presentation of AHA-labeled tumor antigens was assessed using a sandwich ELISA. Significant antigen presentation through MHC class I (**b**) and MHC class II (**c**) was determined by comparing absorbance values to control background levels. Data are presented as mean ± SD. (**, *p* < 0.01, compared to control group).

To further validate our novel assay, we performed ex vivo experiments using bone marrow-derived dendritic cells (BMDCs). Bone marrow stem cells from C57Bl6 mice were differentiated into dendritic cells using GM-CSF and IL-4. Differentiation was confirmed by staining with CD1a and CD11c, established dendritic cell markers, with results showing over 95% of cells expressing both markers (Fig. 6a). BMDCs were then incubated with 0.1 mg of AHA-labeled breast tumor antigens for 6 days. Antigen presentation via MHC I and MHC II was subsequently evaluated using sandwich ELISA. Our findings illustrated that AHA-containing breast tumor antigens can be markedly presented both by MHC I (Fig. 6b) and MHC II (Fig. 6c) in primary dendritic cells when compared with control group. BMDCs with no AHA-labeled tumor antigens were regarded as controls.

## Discussion

Current methodologies to measure antigen presentation rely on pre-defined antigen sequences and antibodies specific to those antigens. Antigen presentation by MHC molecules, including both MHC I and MHC II, is highly selective^30,31^. MHC I presents antigens with hydrophobic ends and specific amino acid side chains that fit into its binding pocket in the central backbone, while MHC II interacts with antigens through specific backbone interactions^32^. These stringent requirements allow antigens to be stably presented by MHC molecules for extended periods, enabling T-cell priming in lymphoid tissues. However, if antigens do not perfectly interact with MHC molecules, antigen editing occurs—in the endo-plasmic reticulum (ER) for MHC I and in endosomes for MHC II—leading to further digestion or complete replacement with alternative antigens. This makes selecting a pre-defined antigen sequence challenging, as no standardized parameters currently exist to identify the optimal sequence for antigen presentation. Consequently, current methods that rely on pre-defined antigens face concerns about the reliability of detecting antigen presentation and the consistency of measuring downstream T-cell activation. These issues arise when pre-defined antigens are modified through editing or dissociating from MHC molecules.

In this study, we introduce a novel assay based on click technology to effectively and reliably measure antigen presentation in RAW264.7, DC2.4 and BMDCs. Click chemistry is known as the reaction between alkyne/cyclooctyne and azide^28^. This reaction has a safety profile in living systems and a huge potential for in vitro applications in the presence of living cells^33^. DBCO, a cyclooctyne, is thermostable with highly specific reactivity toward azides, resulting in quantitative yields of stable triazoles. These features make DBCO ideal for investigating and measuring the entire process of antigen presentation in living APCs. Intracellularly, phagolysosomes are acidic compartments where engulfed exogenous antigens are digested into antigenic peptides before being loaded onto MHC II molecules^34^. Our findings reveal that DBCO-labeled N-terminus tumor antigens remain stably localized and are not removed in the acidic phagolysosomes. This suggests that our new method based on click technology can effectively detect the intracellular process of antigen presentation in APCs.

In addition to DBCO as a protein labeling linker, azide can also be used as a protein linker reversely. AHA is modified with azido moiety, which can be detected by fluorescent or biotin-labeled DBCO/alkyne. Given that AHA is an analog of methionine, it can be incorporated into newly synthesized proteins by re-placing methionine during translation^35–37^. This allows full-length proteins to be thoroughly labeled with azide. Our findings demonstrate that AHA exhibits comparable stability in the low pH environment of phagolysosomes. Combined with its specific binding affinity for alkyne/cyclooctyne and its thermostability, AHA proves to be equally efficient as DBCO for studying antigen processing and presentation.

To evaluate our assay, we utilize two types of antigen preparations: homogeneous antigenic peptides derived from BSA protein and heterogeneous protein mixtures derived from bacteria or tumor cells. BSA is commonly used as a model foreign antigen in immunological studies due to its simplicity, stability, and well-defined structure. Fumitaka Sato and colleagues showed that BSA is an effective antigen that is capable of increasing CD11c^+^ pulmonary APC function in vivo^38^. It has been reported that BSA as the antigen can be effectively presented by APCs to activate human CD4^+^ T cells from healthy donor peripheral blood mononuclear cells (PBMCs) through Epstein-Barr virus (EBV)--transformed autologous B cells in ex vivo^39^. Bacterial antigens are often more immunogenic than soluble BSA due to their structure of lipopolysaccharides (LPSs)^40^, especially when live bacteria are used. All antigens are labeled at their N-terminus with DBCO. However, most antigens presented by MHC molecules are not located at the N-terminus, as they are typically processed by peptidases^41^. Our findings indicate that DBCO-labeled heterogeneous antigens are less effective for measuring antigen presentation, likely due to the removal of the N-terminus during antigen processing, which creates the hydrophobic ends required for MHC binding, as previously discussed. While the homogeneous DBCO-BSA antigen demonstrated improved detection of antigen presentation, the results are inconsistent and unstable. This is likely due to antigen editing unless a pre-defined antigen sequence meets all the requirements for stable MHC presentation and altogether avoids the antigen editing process.

In addition to the antigen editing concern, another possible explanation that bacterial antigens derived from E. *coli* in this study show weaker surface antigen presentation by RAW264.7 and DC2.4 than BSA is that we used killed bacterial proteins directly instead of living bacteria, which have been proven to possess more robust immune responses than killed microorganisms in vaccination^42–44^. Moreover, a landmark study demonstrated that vaccination with heat-killed Listeria monocytogenes failed to prime effector CD8^+^ T cells in mice^45^. The autoclaved operation used in this study may change the bacterial particulate nature and associated pathogen-associated molecular patterns (PAMPs) that APCs can easily recognize via pattern recognition receptors (PRRs), leading to their decreased uptake, processing, and presentation efficiency by APCs.

Since terminal labeling with click chemistry molecules can potentially be removed during antigen editing and processing^46^, we propose an alternative approach: incorporating the click chemistry molecule throughout the entire peptide. This ensures that, regardless of how the peptides are cleaved or digested, there is a high likelihood that a click molecule will remain for detection. To achieve this, we utilized AHA as proof of concept. We observe that AHA-labelled tumor antigens exhibit higher levels of surface antigen presentation in both RAW264.7 and DC2.4 compared to DBCO-labeled antigens. Our findings demonstrate that widespread AHA incorporation into heterogeneous antigens improves the assay’s ability to capture the most antigenic or immunodominant epitopes for MHC presentation in APCs, likely due to reduced loss during antigen editing process.

Consistent with what is known by literature, we also observe that DC2.4 cells perform better DBCO/AHA-tumor antigen presentation than RAW264.7 cells. This can be explained by the fact that macrophages typically possess less efficient antigen presentation because of lower MHC II levels than dendritic cells and B cells^47^. Moreover, dendritic cells are the most potent APCs with a primary function for antigen presentation, unlike macrophages and B cells^47,48^.

Antigens presented by different MHC molecules activate distinct subsets of T cells. Specifically, antigens presented via MHC I activate CD8^+^ T cells, while those presented via MHC II activate CD4^+^ T cells. In this study, our proposed assay can distinguish between antigens presented by MHC I and MHC II. Our findings reveal that RAW264.7 and DC2.4 predominantly present antigens via MHC class II on their surface after exposure to tumor antigens, especially with AHA-containing tumor antigens^49^. MHC I has a relatively narrow binding cleft that accommodates shorter peptides (8–11 amino acids), whereas MHC II possesses a more open binding pocket, allowing it to bind longer peptides (13–25 amino acids)^50^. Since antigen recognition in our assay relies on the incorporation of AHA, the extended peptide length and broader binding capacity of MHC II may facilitate enhanced detection. Additionally, it is also possible that this increased detection on MHC II is due to the uptake of tumor antigens via phagocytosis. Once internalized, exogenous antigens are processed in the phagolysosome, where MHC II is located. Since the DBCO-/AHA-tumor antigens used in this study are exogenous, RAW264.7 and DC2.4 primarily present antigens by MHC II instead of MHC I. This aligns with established knowledge that MHC II molecules on APCs present extracellular peptides, while MHC I molecules present intracellular peptides^51,52^.

This study also observed a significant level of cross-presentation of exogenous antigens by MHC I. Since MHC I presentation activates CD8^+^ T cells, cross-priming of exogenous antigens through MHC I plays a critical role in eliciting cytotoxic CD8^+^ T-cell response. Several mechanisms facilitating cross-priming by MHC I have been reported. For example, phagolysosome rupture can release antigens into the cytosol, which are transported to the ER for loading onto MHC I^53^. Additionally, membrane-bound MHC I can undergo recycling, allowing its delivery to endosomes for antigen loading^54^. Although cross-presentation is typically a feature of some primary dendritic cell subsets, existing literature also demonstrates that both RAW264.7 and DC2.4 can cross-present exogenous antigens via MHC I molecules^55^. Interestingly, RAW264.7 and DCs exhibit greater detection of cross-presented antigens when exposed to AHA-tumor antigens compared to DBCO-labeled tumor antigens. This is likely due to the broader incorporation of AHA throughout the antigen, in contrast to DBCO, which is labeled at the N-terminus and may be cleaved by peptidases during cytosolic processing in cross-presentation. Furthermore, using primary dendritic cells derived from mouse bone marrow, we demonstrated that AHA-incorporated tumor antigens can be presented through both MHC I and MHC II.

As tumors progress, it has been reported that tumor cells may downregulate MHC I expression and reduce the expression of certain tumor antigens, enabling them to evade recognition by the immune system^56–58^. Furthermore, a single-defined peptide may not be representative of all tumor cells due to tumor heterogeneity. Therefore, relying on a pre-defined tumor antigen as the sole measure of antigen presentation and subsequent immune activation may not be practical or accurate. In our developed assay, AHA can be incorporated into peptides derived from whole tumor lysates. This enables APCs to present a diverse array of tumor antigens, even if specific antigens are downregulated in certain tumor cells, providing a more accurate representation of in vivo conditions.

Our developed assay leverages the advantages of “Click-chemistry” technology, which is fast, sensitive, non-toxic, nonradioactive, and time-efficient. In this study, we propose the use of AHA, a methionine analog, for antigen detection. Literature reports indicate that methionine is relatively rare in peptides, with an average frequency of approximately 2.3%^59^. This low abundance may partially explain the limited antigen presentation observed on the surface of DC2.4 cells. However, other amino acids with higher prevalence in peptides, such as alanine (7.4%), lysine (7.2%), and phenylalanine (4.0%), can also be chemically modified for click chemistry applications. In this work, AHA was used as proof of concept, demonstrating the feasibility of this approach. Future strategies incorporating a combination of such modified amino acids could enhance labeling efficiency and improve overall incorporation into antigens. Additionally, all data in this study are collected from in vitro experiments. Future in vivo or ex vivo experimentations such as subsequent T cell activation are needed to validate the reliability and efficacy of this novel assay.

In conclusion, our novel assay system successfully detects antigen presentation on the surface of APCs. Moreover, the fully azido/DBCO-labeled proteins exhibit higher antigen presentation efficiency than the N-terminal azido/DBCO-labeled proteins. This study also addresses the limitations of current antigen presentation measurement by introducing a rapid and user-friendly examination system based on click chemistry. It allows for earlier and more precise detection of functional antigen presentation and potential immune responses. For example, the presentation of click chemistry–modified peptide vaccines can be detected within 1–3 days, offering a rapid means to assess vaccine efficacy—significantly faster than the conventional 21–28 day wait required for antibody titer measurements. As such, this assay holds promise for advancing both diagnostic and therapeutic strategies across a range of diseases, ultimately contributing to improved global health outcomes.

## Methods

### Cell culture

Mouse dendritic cells (DC2.4) (SCC142, Sigma-Aldrich), mouse macrophages (RAW264.7) (91062702, Sigma-Aldrich), and triple-negative mouse breast cancer cells (EO771) (CRL-3461TM, ATCC) were cultured in a standard medium containing RPMI 1640 (Cat. #10-040-CV, Corning) supplemented with 10% fetal bovine serum (FBS, 10082-147, ThermoFisher Scientific) and 1% penicillin/streptomycin supplement (SV30010, Citiva). All cells were cultured at 37 °C in an incubator with 5% CO₂. The DC2.4 cells are derived from the C57BL/6 mouse strain, while the RAW264.7 cells originate from the BalbC/J strain. This study utilized both cell types not only as two distinct antigen-presenting cells (APCs) but also to represent antigen presentation across two different mouse genetic backgrounds.

Mouse bone marrow cells from wild-type C57BL/6 mice were generously provided by a colleague at Texas Tech University (acknowledged accordingly), with collection performed under approved IACUC protocols at that institution. The cells were differentiated into dendritic cells by culturing them with 50 ng/mL GM-CSF (10787-940, VWR) and 50 ng/mL IL-4 (103622-832, VWR) for 7 days. The identity of the resulting dendritic cells was confirmed by the expression of CD11c-FITC (11-0114-82, ThermoFisher) and CD1a-PE (12-0019-4, ThermoFisher), both established markers of dendritic cells.

### Antigen preparations

*E.* *coli* (EC0114, ThermoFisher Scientific) was cultured in serum-free RPMI1640 medium at room temperature for 3–4 days until the culture medium became turbid. It was then autoclaved at 131 ℃ for 30 min. Bacterial proteins derived from E. coli were collected, and their concentration was measured using Bradford Assay (34029, ThermoFisher Scientific). Every 0.1 mg prepared *E.* *coli* protein was reacted with 4 × 10^4^ µM of DBCO crosslinker (C20039, ThermoFisher Scientific) for 2 h on ice to form DBCO-conjugated *E.* *coli* antigen. Unbound DBCO was filtered out using the columns (PIA44297, ThermoFisher Scientific).

BSA lyophilized powder (84-2251, Carolina Biological) was thoroughly dissolved in sterile ddH2O, and a 0.22-µm filter was used to filter out the non-dissolved deposits and potential contaminants in the sterile cabinet. Bradford Assay analyzed the dissolved BSA concentration. The same DBCO conjugation ratio (0.1 mg BSA: 4 × 10^4^ µM DBCO) was applied, and unbound DBCO was filtered out using columns.

Tumor antigens were prepared using two distinct methods. In the first method, EO771 cells were cultured until reaching 80–90% confluency. Tumor cell lysates were then collected for protein extraction using cell lysis buffer (87788, ThermoFisher Scientific). After extraction and quantification, tumor proteins were conjugated with DBCO at a ratio of 0.1 mg tumor protein to a specified concentration of DBCO (4 × 10^4^ µM). Un-bound DBCO was removed using filtration columns. However, since DBCO interacts with amines (N-terminus) and might be removed during antigen processing by UPS, potentially resulting in signal loss, a second method was employed. In the second method, EO771 cells were cultured in a complete RPMI 1640 medium supplemented with L-Azidohomoalanine-hydrochloride (L-AHA; MSPP-CS0120777, VWR). L-AHA, an amino acid analog of methionine, is incorporated into peptides as a methionine substitute. This integration ensures that processed antigens retain the azide group during ER antigen editing, increasing the likelihood of successful detection via click chemistry.

### Measurement of phagocytosis

To evaluate whether DBCO conjugation or AHA integration affects phagocytosis by macrophages, RAW264.7 were cultured on chamber slide (1 × 10^5^ cells/slide) and exposed to pHrodo Green-crosslinked DBCO- and AHA-containing tumor antigens (0.1 mg) for 1 day, respectively. The pHhrodo™ Green STP ester (Cat. #P35369) is a dye that fluoresces green at low pH environments (such as phagolysosome). Cells were also stained with nuclear dye (blue, R37609, ThermoFisher Scientific) and cell membrane-permeable azide (FITC, red). Cells treated with pHrodo-conjugated AHA-containing tumor fragments were stained with nuclear dye (DAPI, blue) and cell membrane-permeable alkyne (red). All staining were performed for 30 min at 37 ℃. Immunofluorescence images were acquired using Revolve R4 microscope (RVSF1000, Echo). Quantitative analysis of mean fluorescence intensity from immunofluorescence images was performed using ImageJ software (version 13.0.6).

### Measurement of surface antigen presentation using fluorescent microplate reader

DC2.4 and RAW264.7 were cultured in 96-well microplates (1,000 cells/well) to assess the antigen-presenting capacity. Cells were exposed to three types of DBCO-conjugated antigens (*E. coli*, BSA, and tumor peptides) for 6 days in an incubator at 37℃, with 6 replicates per group. After 6 days, the old media was removed. Cells were stained with impermeable Alexa Fluor™ 488 azide (C10428, ThermoFisher Scientific) at a concentration of 1.6 µl/ml for 30 min at 37 ℃. The impermeable Alexa Fluor™ 488 azide specifically reacts with DBCO-conjugated antigens on the cell surface, and the resulting surface fluorescent intensity of DC2.4 and RAW264.7 was determined using Synergy H1 microplate reader machine (2208230B, Biotech) with excitation 450 nm, and emission 520 nm.

### Examination of surface antigen presentation via MHC class I or class II using enzyme-linked immunosorbent assay (ELISA)

While antigens acquired from phagocytosis are typically presented through MHC II, it has been shown that cross-presentation through MHC I also occurs^60^. MHC I presents antigens that are hydrophobic on both ends with special side chains that fit the surface-presenting pocket^61^. To determine if the DBCO conjugation and/or AHA integration would impact the antigen presentation on MHC class I or II, an ELISA was performed.

RAW264.7 were treated with DBCO- or AHA-containing tumor protein for 6 days. Cells were lysed using a lysis buffer, and cellular proteins were harvested for sandwich ELISA analysis. Anti-mouse MHC class I (for MHC I of macrophages from BalbC/J mice of H-2K^d^ haplotype, 116601, Biolegend), anti-mouse MHC class I (for MHC I of DC2.4 from C57BL/6 mice of H-2K^b^ haplotype, 116502, Biolegend), and anti-mouse MCH class II (70-5321-U100, TONBO Biosciences) at a concentration of 1 µg/ml were pre-coated on ELISA plate at 4 ℃ overnight. After washing the plate 3 times with wash buffer (Phosphate Buffered Saline (PBS) containing 0.05% weight/volume of Tween-20), we added 150 µL of blocking solution (Phosphate Buffered Saline (PBS) containing 5% weight/volume of BSA) to each well at 4 ℃ overnight. After 4 times washing, sample proteins with a concentration of 0.5 µg/ml from RAW264.7 and DC2.4 were added and incubated for 1 h at 37℃. After 3 times in wash buffer, biotin-conjugated azide (3020, AAT Bioquest) at a concentration of 50 ng/ml was added into the DBCO-labeled tumor antigen group, while biotin-conjugated alkyne (3021, AAT Bioquest) at a concentration of 50 ng/ml was added into the AHA-labeled tumor antigen group. Then the plate was incubated at 37℃ incubator for 1 h. Next, 100 µL of HRP-conjugated streptavidin (#405210, AAT Bioquest) at a concentration of 50 ng/ml at 37℃ incubator for 30 min after 3 times wash. Then, 100 µL of TMB substrate solution (#34029, ThermoFisher Scientific) was added to each well at room temperature and in the dark for 20 min until color change happened in the wells. Finally, we put 100 µl of stop solution (#SS04, ThermoFisher Scientific) into each well and read absorbance at 595 nm using a Bio-Rad (Hercules, CA) model 550 microplate reader. All samples, negative and positive groups, were in triplicate.

### Flow cytometry

To compare fluorescent DBCO-/AHA-tumor antigen presentation by APCs, RAW264.7 and DC2.4 were seeded in 6-well plates (5 × 10^5^ cells/ml) and treated with or without DBCO/AHA-tumor antigens under standard cell culture conditions for 6 days. RAW264.7 and DC2.4 were detached using 1 × trypsin and centrifuged at 1,500 rpm for 5 min at room temperature. Then, cells were resuspended in 100 µL of DPBS and were added to the Fc block for 5 min. After washing in DPBS, the samples were resuspended in 200 µL of DPBS. Cells treated with DBCO-labeled tumor antigens were incubated with biotin-azide at an amount of 0.4 µg. In contrast, samples treated with AHA-containing tumor antigens were incubated with biotin-alkyne at an amount of 0.4 µg for 30 min at 4 ℃. After washing in DPBS, APCs surface staining was performed using a fluorescently conjugated anti-biotin antibody (Cat. # 53-9895-82, ThermoFisher Scientific) at an amount of 0.4 µg for 30 min at 4 ℃. Staining APCs were analyzed using flow cytometry (BSN-ZS-AS7S, Conduct Science). Unstained RAW264.7 or DC2.4 served as negative controls.

### Statistical analysis

The statistical significance was determined using a two-tailed paired Student’s *t*-test for comparison between the two groups. All graphs were generated using Prism 9.1.1-Graphpad. All data are presented as means *±* SD. All experiments were performed at least three times unless otherwise noted in the figure legend. A P value of < 0.05 was considered significant.

## Supplementary Information

Below is the link to the electronic supplementary material.

Supplementary Material 1

## Data Availability

The dataset supporting the conclusions of this article is available from the corresponding author.
