# Supplementary material for: Development of a novel assay for antigen presentation measurement
Source: Sci Rep. 2025 Sep 26;15:33168. doi: 10.1038/s41598-025-13997-y (PMC12475488; doi:10.1038/s41598-025-13997-y)
Supplement: Supplementary file 1 — Supplementary Material 1 [file 41598_2025_13997_MOESM1_ESM.docx]

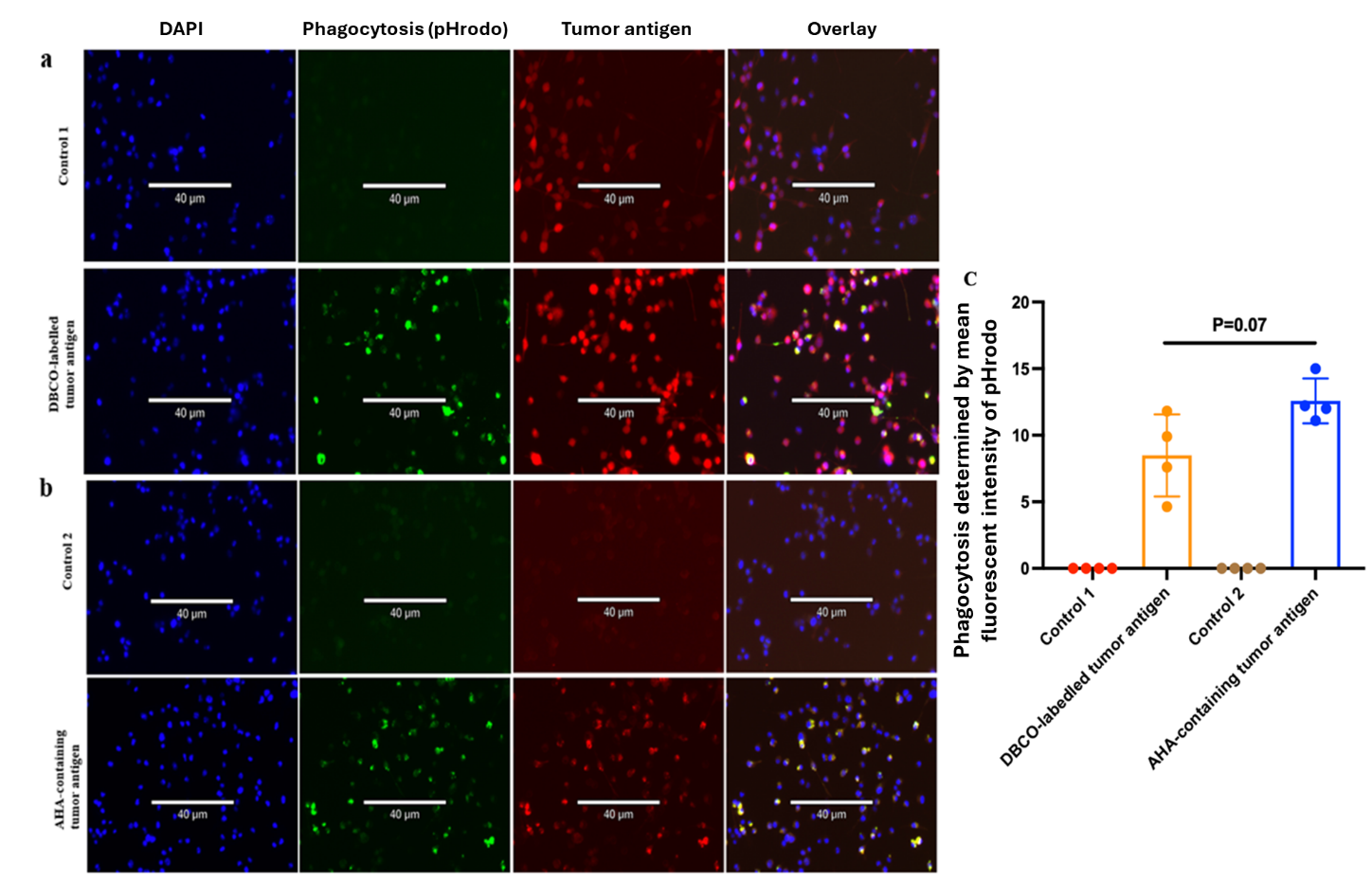


**Supplementary Figure 1: The azido/alkyne-labeled antigens are stable in the phagolysosomes of dendritic cells (a and b).** Triple immunofluorescence staining is used to examine the phagocytosis of DBCO-labelled tumor antigens (a) and AHA-containing tumor antigens (b) by dendritic cells. The blue, green, and red fluorescence labels the nucleus, phagolysosome, and DBCO-labeled/AHA-containing tumor antigens. Dendritic cells treated without any tumor antigens were used as controls. The mean fluorescence intensity of the green signal, indicative of phagocytosis, was quantified (c). Data are shown as mean ± SD. DBCO, dibenzocyclooctyne; AHA, L-Azidohomoalanine.
